# Notes and Notices

**Published:** 1893-04

**Authors:** 


### NOTES AND NOTICES.

The Statue of Hahnemann.—The joint meeting of the committee appointed by the State Homœopathic Society of Pennsylvania and the County Homœopathic Society of Philadelphia took place at the Hahnemannian Medical College of Philadelphia, April 6th, 1893, at four P. M., to devise means by which funds may be raised for the erection of the Hahnemann statue at Washington, D. C.

Dr. Jas. H. McClelland, of Pittsburgh, called the meeting to order and made an address commemorative of Hahnemann.

Dr. A. R. Thomas was appointed Chairman *pro tem.*

Dr. Aug. Korndœrfer was elected Chairman of the State Committee; Dr. Bushrod W. James, Treasurer; Dr. C. S. Schwenk, Secretary.

The greatest amount of interest has been manifested by the homœopathic profession in this matter. Thousands of dollars have already been subscribed and if we are to judge from the well-known liberality of the homœopathic physicians of the United States, a statue will be erected at our capital which will not only honor the discoverer of the rational and scientific system of therapeutics and materia medica, but it will be regarded with pride by a nation which has felt the benign influence of Hahnemannian teachings.

Dr. Henry M. Smith, of New York City, Secretary and Treasurer of the American Institute of Homœopathy, was present, and gave a most flattering report of the number of contributions that he has received from all directions. Everybody is giving as freely as their means will permit, from $1.00 up to $100.00.

Some valuable suggestions were offered by Dr. Jos. E. Jones, of West Chester, Pa., and Dr. Jos. C. Guernsey, of Philadelphia, after which, at 6.30 P. M., the meeting was adjourned. C. S. SCHWENK, *Secretary.*

THE WORLD'S CONGRESS OF HOMŒOPATHIC PHYSICIANS AND SURGEONS AT CHICAGO, MAY 29TH, 1893.—Arrangements for the Congress are being rapidly completed. Some of the addresses have been received. All the Chairmen of Sections are actively at work, and report marked progress. Several Sections are completed and the rest will soon be in order. All the papers will be of high character. Many of the veterans in the profession will be present and deliver addresses on subjects of vital interest. In the scientific work of the Congress, the younger men who have achieved distinction in our school will be represented. The sections which they conduct will be made very interesting through their work and that of their worthy associates. Women will be ably represented in all departments. There is every prospect that the Congress will assume a pronounced international character.

The eyes of the world will be upon Chicago during the Exposition period. The proceedings of the Congress will be universally and fully reported. Every homœopath laboring in his national, state, or local society, will find his work made easy by the results of a convention of grand proportions and sterling work.

Let every physician of our school make a sacrifice if necessary, to be present.

J. S. MITCHELL, M. D.,
*Chairman World's Congress Homœopathic Physicians and Surgeons.*

J. P. DAKE, M. D.,
*Chairman American Institute Committee on World's Congress.*

THE COMMITTEE ON ARRANGEMENTS for the World's Congress of Homœopathic Physicians, announces that it has made arrangements with different hotels and apartment houses to accommodate at least 2,500 guests. If notified in time it can take care of fully double this number.

The Chairman of the Committee, Dr. A. K. Crawford, 70 State Street, Chicago, wishes it distinctly understood that unless he is applied to prior to the meeting of the Congress, he will not be responsible for accommodations of intended visitors.

The prices arranged for range from $1.00 per day and up, European plan; and $2.50 per day and up, American plan.

THE INTERNATIONAL HAHNEMANNIAN ASSOCIATION will hold its next meeting at Kaye's Park, Lake Geneva, Wis., June 7th, 8th, and 9th. Hotel rates, $2.00 per day. Kaye's Park is two and one-half hours' ride from Chicago, on the "Chicago & Northwestern Railroad." It is one of the most beautiful summer resorts of Wisconsin, and the short distance from Chicago will be of great convenience in visiting the World's Fair, either before or after the meeting. We should have a large attendance, and all who intend to visit Chicago should make arrangements to attend the meeting. Members are earnestly requested to send the titles of their papers to the Secretary at an early date, that the "Order of Business" may be as complete as possible. Circulars descriptive of Kaye's Park will be sent later. Very truly yours, Samuel A. Kimball, Secretary, 124 Commonwealth Avenue, Boston, Mass.

THE MISSOURI INSTITUTE of Homœopathy will hold its Seventeenth Annual Session in Kansas City, Tuesday, Wednesday, and Thursday, April 18th, 19th, and 20th next, at Hotel Midland.

DR. CHARLES B. GILBERT has resumed the practice of medicine at 1403 H Street, N. W., Washington, D. C.

AN AMERICAN TEXT-BOOK OF THE DISEASES OF CHILDREN, including special chapters on Essential Surgical Subjects; Diseases of the Eye, Ear, Nose, and Throat; Diseases of the Skin; and on the Diet, Hygiene, and General Management of Children, by American teachers. Edited by Louis Starr, M. D., assisted by Thompson S. Wescott, M. D., forming a handsome imperial 8vo volume of about one thousand pages, profusely illustrated. For sale by subscription only. W. B. Saunders, 913 Walnut Street, Philadelphia. Now ready Volume I, *American Text-Book of Practice*, edited by Dr. William Pepper.

GRACE HOSPITAL of Detroit, Michigan, recently submitted its annual report showing an annual expenditure of $43,065.51. Total revenue, $41,701.61, leaving a deficiency of $1,363.90. The number of patients treated in the Hospital was 1,094, in the Dispensary 4,870. Average cost per person per day 83 3-10 cents. Further details may be had by applying to the Superintendent, Mr. Robert N. Silliman.

THE MEDICAL ADVANCE offers special inducements to new subscribers. See its offer in the advertising pages of this journal.

A COMPETITIVE EXAMINATION of Resident and Associate-Resident Physicians of the Children's Homœopathic Hospital of Philadelphia will be held at the Hospital, 926 North Broad Street, on Tuesday, April 18th, at 12 o'clock noon. Applications can be sent to Dr. Jas. M. Reeves, President of the Medical Board, or to Dr. Bushrod W. James, President of the Hospital.

THE INTERNATIONAL CONGRESS OF CHARITIES, CORRECTION, AND PHILANTHROPY makes one of the series of International Congresses to be held in Chicago in 1893. The fourth Section of this is to consider all matters relating to the Hospital Care of the Sick, the Training of Nurses, Dispensary Work, and First Aid to the Injured. The Committee of Organization of the Congress has appointed Dr. John S. Billings, Surgeon U. S. Army, as Chairman of this Section, and Dr. Henry M. Hurd, Superintendent of the Johns Hopkins Hospital in Baltimore, as its Secretary, and has authorized and requested them to complete its organization.

Persons desiring to present papers, or to share in the discussions of this Section, are requested to communicate with the Secretary at once. The period of time allotted for the preparatiou of the programme is necessarily brief, and it is essential that all who are willing to assist in this work should act promptly· Address all communications to Dr. Henry M. Hurd, The Johns Hopkins Hospital, Baltimore, Md.

Annual Reunion of the Alumni Association of the Hahnemann Medical College, Philadelphia, April 19th, 1893.—The Alumni Association of the Hahnemann Medical College, Philadelphia, requests the pleasure of the company of the Alumni of the College, at its Annual Re-union and Banquet, on Wednesday, April 19th, 1893.

The Business Meeting will convene at 4.30 P. M. in Alumni Hall, Hahnemann Medical College, Broad Street above Race, Philadelphia, and the banquet will be held at 10 P. M. at "The Stratford," corner of Broad and Walnut Streets.

The Trustees and Faculty of the College extend a cordial invitation to all the members of the Alumni and their friends to attend the Forty-fifth Annual Commencement to be held on the same evening, at eight o'clock, at the Academy of Music, Broad and Locust Streets, Philadelphia.

Banquet Cards can be secured from any officer of the Association, at $3.50 each. The cards being limited to two hundred, the Committee cannot guarantee to furnish any applied for after April 18th, 1893. If you can make arrangements to be present at the banquet, notify the Secretary and he will secure a place for you. W. W. Van Baun, M. D., *Secretary.*
419 Pine Street, Philadelphia, Pa

Doctor Patch wishes to announce to his patrons that he will be absent from town during the months of March, April, and May, for the purpose of clinical study at Dublin and London.

During this time his practice will be conducted by Dr. R. A. Davis, a thoroughly competent physician, who will occupy the same office, have access to all past case records, and, we trust, be able to give every satisfaction to those who may need his services. South Framingham, February 15th, 1893.

Many physicians are recommending the use of Horlick's Malted Milk as a table drink in place of tea, coffee, cocoa, etc. The evil effects of long-continued use of tea or coffee are well-known, but the difficulty has been to provide a pleasant and satisfactory subsbitute. Malted Milk is a perfect soluble combination of pure cows' milk and an extract of malted grain, and, when served either hot or iced, it makes one of the most pleasant, refreshing, and nutritious drinks imaginable, little if any more expensive than the ordinary drinks, and far more healthy and nutritious for continued use. Does not stimulate, but aids digestion. Prepared by simply adding water. Address the Malted Milk Co., Racine, Wis., for samples.

